# Whole-exome sequencing identified genetic risk factors for asparaginase-related complications in childhood ALL patients

**DOI:** 10.18632/oncotarget.17959

**Published:** 2017-05-17

**Authors:** Rachid Abaji, Vincent Gagné, Chang Jiang Xu, Jean-François Spinella, Francesco Ceppi, Caroline Laverdière, Jean-Marie Leclerc, Stephen E. Sallan, Donna Neuberg, Jeffery L. Kutok, Lewis B. Silverman, Daniel Sinnett, Maja Krajinovic

**Affiliations:** ^1^ Research Center, Centre Hospitalier Universitaire Sainte-Justine, Montreal, QC, Canada; ^2^ Department of Pediatrics, University of Montreal, Montreal, QC, Canada; ^3^ Department of Pediatric Oncology, Dana-Farber Cancer Institute, Boston, MA, USA; ^4^ Division of Hematology/Oncology, Children’s Hospital, Boston, MA, USA; ^5^ Department of Biostatistics and Computational Biology, Dana-Farber Cancer Institute, Boston, MA, USA; ^6^ Department of Pathology, Brigham and Women’s Hospital, Boston, MA, USA; ^7^ Department of Pharmacology, University of Montreal, Montreal, QC, Canada

**Keywords:** acute lymphoblastic leukemia, asparaginase, exome-wide association, pharmacogenetics, whole-exome sequencing

## Abstract

Allergy, pancreatitis and thrombosis are common side-effects of childhood acute lymphoblastic leukemia (ALL) treatment that are associated with the use of asparaginase (ASNase), a key component in most ALL treatment protocols. Starting with predicted functional germline variants obtained through whole-exome sequencing (WES) data of the Quebec childhood ALL cohort we performed exome-wide association studies with ASNase-related toxicities. A subset of top-ranking variants was further confirmed by genotyping (*N*=302) followed by validation in an independent replication group (*N*=282); except for thrombosis which was not available for that dataset. SNPs in 12 genes were associated with ASNase complications in discovery cohort including 3 that were associated with allergy, 3 with pancreatitis and 6 with thrombosis. The risk was further increased through combined SNPs effect (*p*≤0.002), suggesting synergistic interactions between the SNPs identified in each of the studied toxicities. Interestingly, rs3809849 in the *MYBBP1A* gene was associated with allergy (*p*= 0.0006), pancreatitis (*p*=0.002), thrombosis (*p*=0.02), event-free survival (*p*=0.02) and overall survival (*p*=0.003). Furthermore, rs11556218 in *IL16* and rs34708521 in *SPEF2* were both associated with thrombosis (*p*=0.01 and *p*=0.03, respectively) and pancreatitis (*p*=0.02). The association of SNPs in *MYBBP1A, SPEF2* and *IL16* geneswith pancreatitis was replicated in the validation cohort (*p* ≤0.05) as well as in combined cohort (*p*=0.0003, *p*=0.008 and *p*=0.02, respectively). The synergistic effect of combining risk loci had the highest power to predict the development of pancreatitis in both cohorts and was further potentiated in the combined cohort (*p*=1×10^-8^). The present work demonstrates that using WES data is a successful “hypothesis-free” strategy for identifying significant genetic markers modulating the effect of the treatment in childhood ALL.

## INTRODUCTION

Acute lymphoblastic leukemia (ALL) is the most common cancer in children and it accounts for 25% of all childhood malignancies. [1–3] Survival rates have improved significantly over time with the progressive intensification of ALL treatment and the implementation of multi-agent risk-adapted protocols. [2–4] However, a subset of patients experience treatment failure or short-term treatment-related toxicities which might result in the interruption or discontinuation of chemotherapy or can have severe, fatal, or lifelong consequences that challenge their ability to lead a normal life as future adults. [2]

Asparaginase (ASNase) was introduced as major component of ALL treatment protocols in 1970 and has been a mainstay of therapy ever since. [1–3, 5] It is an enzyme that catalyzes the hydrolysis of the amino acid asparagine (ASN) into aspartic acid and ammonia and is thus required by all cells. Cancerous lymphoblasts usually depend on extracellular sources of asparagine to support their fast growth as they have ASNS levels that are relatively lower than their needs. Thus, depletion of asparagine by ASNase reduces the capacity of protein biosynthesis in leukemia cells which selectively promotes their death. [1, 2]

Less favorable outcome in childhood ALL treatment has been associated with treatment discontinuation and the failure to receive the full course of ASNase due to treatment-related toxicities. [2, 4, 6] L-asparaginase comes from 2 bacterial sources, *Escherichia coli (E.coli)* and *Erwinia chrysanthemi. While E. coli*-derived enzyme generally has higher efficacy, it has been reported to have higher toxicity. [1–3] ASNase-related treatment toxicities mostly include allergic reactions, pancreatitis and thrombotic events frequently associated with discontinuation of asparaginase treatment. [1–4]

Given the bacterial origin of asparaginase, it is not surprising that it is capable of inducing immune reactions *in vivo* as up to 30% of patients experience a hypersensitivity reaction to *E. coli*-derived asparaginase. [1–4, 7] While reported rates vary across literature, clinical and subclinical hypersensitivity reactions are associated with decreased asparaginase activity levels caused by neutralizing antibodies and may be influenced by the asparaginase preparation used, dose intensity, and other medications. [3, 4, 7]

Around 2-18% of patients receiving asparaginase develop pancreatitis which is usually associated with clinical symptoms along with serum amylase and/or lipase elevation reaching more than three times upper-normal limits. [3, 4] While currently known risk factors include intensive treatment and older age, the pathogenesis of asparaginase-induced pancreatitis is not yet fully understood and is thought to occur as a result of an underlying predisposition. [2, 8] Interestingly, unlike with hypersensitivity reactions, the incidence of pancreatitis does not seem to be influenced, at least in some studies, by the formulation of asparaginase used. [3, 4, 8]

Thrombosis, defined as venous and/or arterial thromboembolism, has a higher incidence in paediatric oncology patients and is reported with both *E. coli*- and *Erwinia*-derived asparaginase (mainly due to interference with the hepatic synthesis of coagulation proteins) and has an overall incidence of around 5% according to recent studies. [4, 5] Many factors have been associated with the risk of thrombosis, some related to the disease, others to the treatment (like the dose and duration of asparaginase exposure) as well as to patient specific factors such as older age, female gender, non-O blood group, obesity, inherited prothrombotic states or central venous catheter. [3, 5, 9, 10]

Being able to predict which patients will experience asparaginase-related toxicity and switching them to an alternative asparaginase formulations [4] or a different treatment protocol that does not depend heavily on asparaginase has been shown to yield superior outcomes. [8] Accordingly, using genetic markers for prospective stratification of patients at high risk of developing allergic reactions, pancreatitis or thrombosis has the potential to improve ALL treatment by identifying a patient subgroup which might benefit more from an alternative regimen. [4, 8]

Over the past decade, important advances in sequencing technology have been achieved which not only helped deciphering leukemia specific mutations, [11, 12] but also provided comprehensive information on germline polymorphisms for association studies of complex disease traits and suboptimal treatment responses. [11, 12] Here we present the results of an exome-wide association study (EWAS) that was performed on whole exome sequencing (WES) data obtained from childhood patients who received asparaginase as part of ALL treatment protocol. The results provide an insight on novel pharmacogenetic markers associated with asparaginase related allergic reactions, pancreatitis and thrombosis.

## RESULTS

### Asparaginase-related complications

Twenty-nine patients (9.6%) received a formulation containing *Erwinia* derived asparaginase while the rest received an *E.coli* derived formulation (Table 1). The observed frequencies of the asparaginase-related toxicities ware comparable to those reported in the literature [2, 4, 5, 8]: 15.9% (48) patients developed allergies (with 40 of them having serious systemic reactions while the rest having mixed or local reactions); 5% (15) experienced pancreatitis (12 severe and 3 mild to moderate); and 3.3% (10) had thrombosis. Consequently, and following the treatment protocols guidelines, ALL patients with complications needed treatment modification, either interruption or switch to other types of asparaginase.

**Table 1:** Characteristics of the discovery and the replication cohort

Cohort Characteristics		QcALL	DFCI	*p*-Value
Total Included		302	282	
**Sex**	Female	139 (46%)	129 (45,7%)	1
	Male	163 (54%)	153 (54,3%)	
**WBC**	< 50×10^3^/µL	257 (85,1%)	229 (81,2%)	0,2
	> 50×10^3^/µL	45 (14,9%)	53 (18,8%)	
**Age**	< 10 years	242 (80,1%)	230 (81,6%)	0,7
	≥ 10 years	60 (19,9%)	52 (18,4%)	
**Risk**	Standard	151 (50%)	173 (61,3%)	0,007
	High	151 (50%)	109 (38,7%)	
**Source of Asparaginase**	*E. Coli*	273 (90,4%)	261 (92,6%)	0,4
	*Erwinia*	29 (9,6%)	21 (7,4%)	
**DFCI Protocol**	00-01	111 (36,8%)	187 (66,3%)	6×10^-5^
	95-01	119 (39,4%)	95 (33,7%)	
	91-01	55 (18,2%)	-	-
	87-01	17 (5,6%)	-	

QcALL, Quebec Childhood ALL cohort; DFCI, Dana-Farber Cancer Institute ALL Consortium cohort.

Toxicities in replication cohort had similar frequencies to those of the discovery cohort as there were 20.9% (59) patients with allergies (39 systemic) and 7.4% (21) with pancreatitis (14 severe). Information on thrombosis was not available. The frequency of *Erwinia*-derived asparaginase and *E.coli* formulation was also comparable to the discovery cohort.

#### Association study

**Figure 1:**
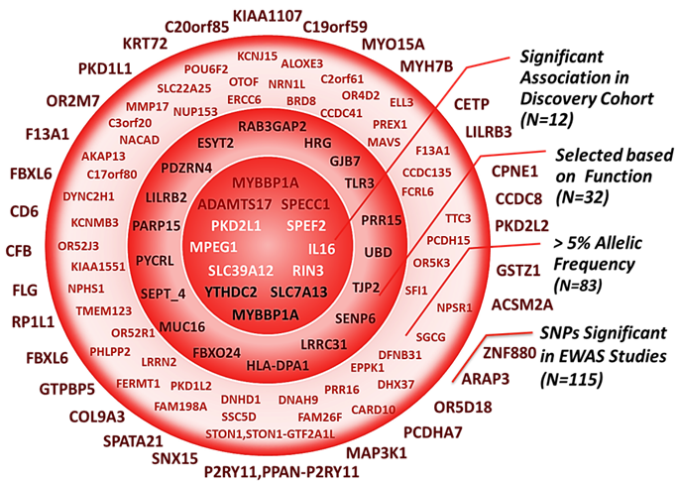
The selection process following the exome-wide association study Top-ranking signals from the EWAS (*N* = 115) were filtered through a multi-step selection process explained on the right-side of the figure. Each circle contains all the SNPs that are inside of it, including the ones in the smaller circles. Inner circle represent significant associations with one of the 3 asparaginase related toxicities (*N* = 12) retained for analysis in replication cohort. rs3809849 in *MYBBP1A* was significantly associated both with allergy and pancreatitis in the EWAS study.

The number of predicted functional common variants recovered from WES data was 5527; from these, 4519 SNPS distributed across 3802 genes, respected Hardy-Weinberg equilibrium and were tested for an association with asparaginase-related toxicities. Out of the 115 top-ranking SNPs identified from WES data with FDR < 20%, 43 were associated with allergy, 40 with pancreatitis and 32 with thrombosis (Supplemental Table S1). Given the relatively large number of hits, selective exclusion was performed to remove the SNPs found in genes that are unlikely to be involved in the pathways of studied toxicities (e.g. genes of the olfactory receptors family and other neurosensory functions as well as the ones whose expression is restricted to tissues that are irrelevant to the toxicity in question). Accordingly, and out of the remaining pool, thirty two SNPs (8 SNPs associated with allergy, 10 with thrombosis and 14 with pancreatitis) with minor allele frequency higher than 5% in discovery cohort and located in genes whose biological function could be relevant to the studied response, were selected (Figure 1 and Supplemental Table S2).

Based on genotyping results, 3 variants were associated with allergy (Table 2). Carriers of the minor allele of rs9656982 in the *SLC7A13* gene and of rs3809849 in the *MYBBP1A* gene were associated in additive manner (OR = 2.1; 95% CI, 1.1-3.9; *p* = 0.02 and OR = 2.4; 95% CI, 1.4-3.9; *p* = 0.0006, respectively), whereas the effect of rs75714066 minor allele in the *YTHDC2* gene followed the dominant model (OR = 3.1; 95% CI, 1.4-7.0; *p* = 0.008).

**Table 2:** Top-ranking signals from the exome-wide association study confirmed by genotyping

Toxicity	Gene_SNP Genotype	Complication		OR (95%-CI)	*P*	Model	Complication		OR (95%-CI)	*P*
		+	-				+	-		
**Allergy**	SLC7A13_rs9656982: A > G*									
	**AA**	37 (77,1%)	217 (87,2%)	1	1				2,1 (1,1-3,9)	**0,02**
	**AG**	8 (16,7%)	30 (12,1%)	1,6 (0,7-3,7)	0,3					
	**GG**	3 (6,3%)	2 (0,8%)	8,8 (1,4-54,5)	0,03					
	MYBBP1A_rs3809849: G > C*									
	**GG**	20 (41,7%)	160 (65%)	1	1				2,4 (1,4-3,9)	**6×10**^-4^
	**GC**	23 (47,9%)	79 (32,1%)	2,3 (1,2-4,5)	0,01					
	**CC**	5 (10,4%)	7 (2,9%)	5,7 (1,7-19,7)	0,01					
	*Y****THDC2_rs75714066: G > C***									
	**GG**	37 (77,1%)	232 (91,3%)	1	1	**GG**	37 (77,1%)	232 (91,3%)	1	*-*
	**GC**	11 (22,9%)	21 (8,3%)	3,3 (1,5-7,4)	0,005	**GC+CC**	11 (22,9%)	22 (8,7%)	3,1 (1,4-7,0)	**0,008**
	**CC**	0 (0%)	1 (0,4%)	NA	-					
**Pancreatitis**	***ADAMTS17_rs72755233: G > A***									
	**GG**	7 (46,7%)	232 (83,2%)	1	1	**GG**	7 (46,7%)	232 (83,1%)	1	**-**
	**GA**	8 (53,3%)	45 (16,1%)	5,9 (2-17,1)	0,002	**GA+AA**	8 (53,3%)	47 (16,9%)	5,6 (1,9-16,3)	**0,002**
	**AA**	0 (0%)	2 (0,7%)	NA	-					
	***MYBBP1A_rs3809849: G > C***									
	**GG**	3 (20%)	177 (63,4%)	1	1	**GG**	3 (20%)	177 (63,4%)	1	**-**
	**GC**	12 (80%)	90 (32,3%)	7,9 (2,2-28,6)	0,0005	**GC+CC**	12 (80%)	102 (36,6%)	6,9 (1,9-25,2)	**0,002**
	**CC**	0 (0%)	12 (4,3%)	NA	-					
	SPECC1_rs9908032: C > G*									
	**CC**	8 (53,3%)	228 (80,6%)	1	1				3,9 (1,6-9,2)	**8×10-4**
	**CG**	5 (33,3%)	53 (18,7%)	2,7 (0,8-8,5)	0,1					
	**GG**	2 (13,3%)	2 (0,7%)	28,5 (3,6-228,8)	0,009					
**Thrombosis**	***PKD2L1_rs6584356: C > A***									
	**CC**	7 (70%)	257 (92,1%)	1	1	**CC**	7 (70%)	257 (92,1%)	1	**-**
	**CA**	2 (20%)	22 (7,9%)	3,3 (0,7-17)	0,2	**CA+AA**	3 (30%)	22 (7,9%)	5 (1,2-20,7)	**0,05**
	**AA**	1 (10%)	0 (0%)	NA	-					
	***RIN3_rs3742717: C > T***									
	**CC**	6 (60%)	219 (77,7%)	1	1	**CC+CT**	8 (80%)	277 (98,2%)	13,8 (2,3-82,5)	**0,02**
	**CT**	2 (20%)	58 (20,6%)	1,3 (0,2-6,4)	1					
	**TT**	2 (20%)	5 (1,8%)	14,6 (2,3-91)	0,02	**TT**	2 (20%)	5 (1,8%)		
	***SPEF2_rs34708521: G > A***									
	**GG**	5 (62,5%)	242 (91%)	1	1	**GG**	5 (62,5%)	242 (91%)	1	**-**
	**GA**	3 (37,5%)	23 (8,7%)	6,3 (1,4-28,1)	0,03	**GA+AA**	3 (37,5%)	24 (9%)	6,1 (1,4-26,9)	**0,03**
	**AA**	0 (0%)	1 (0,4%)	NA	-					
**Thrombosis**	SLC39A12_rs62619938: C > T*									
	**CC**	6 (60%)	262 (91%)	1	1				4,4 (1,6-11,7)	**5×10**^-4^
	**CT**	3 (30%)	23 (8%)	5,7 (1,3-24,3)	0,04					
	**TT**	1 (10%)	3 (1%)	14,6 (1,3-161)	0,1					
	***MPEG1_rs7926933: G > A***									
	**GG**	4 (44,4%)	234 (82,1%)	1	1	GG	4 (44,4%)	234 (82,1%)	1	**-**
	**GA**	5 (55,6%)	45 (15,8%)	6,5 (1,7-25,1)	0,009	GA+AA	5 (55,6%)	51 (17,9%)	5,7 (1,5-22,1)	**0,01**
	**AA**	0 (0%)	6 (2,1%)	NA	-					
	***IL16_rs11556218: T > G***									
	**TT**	4 (50%)	238 (88,2%)	1	1	TT	4 (50%)	238 (88,1%)	1	**-**
	**TG**	4 (50%)	30 (11,1%)	7,9 (1,9-33,4)	0,009	TG+GG	4 (50%)	32 (11,9%)	7,4 (1,8-31,2)	**0,01**
	**GG**	0 (0%)	2 (0,7%)	NA	-					

The SNPs are presented as a change from major to minor alleles. OR, odds ratio; CI, confidence interval. Analysis in both co-dominant model and a model that best fits the data are presented. The final models are either dominant, recessive or additive; the latter is indicted by asterisk. NA, not analyzed due to low numbers.

Three SNPs were significantly associated with a risk of pancreatitis (Table 2). Carriers of the minor allele of rs72755233 in the *ADAMTS17* gene and of rs3809849 in the *MYBBP1A* gene were at higher risk of pancreatitis when compared to non-carriers (OR = 5.6; 95% CI, 1.9-16.3; *p* = 0.002 and OR = 6.9; 95% CI, 1.9-25.2; *p* = 0.002, respectively), whereas the SNP (rs9908032) in the *SPECC1* gene followed the additive model (OR = 3.9; 95% CI, 1.6-9.2; *p* = 0.0008).

Six SNPs were associated with thrombosis (Table 2). Carriers of minor alleles were predisposed to a higher risk when compared to non-carriers including rs6584356 in *PKD2L1* (OR = 5.0; 95% CI, 1.2-20.7; *P* = 0.05); rs3742717 in *RIN3* (OR = 13.8; 95% CI, 2.3-82.5; *P* = 0.02); rs34708521 in *SPEF2* (OR = 6.1; 95% CI, 1.4-26.9; *P* = 0.03); rs7926933 in *MPEG1* (OR = 5.7; 95% CI, 1.5-22.1; *P* = 0.01); rs11556218 in *IL16* (OR = 7.4; 95% CI, 1.8-31.2; *P* = 0.01) and rs62619938 in *SLC39A12* (OR = 4.4; 95% CI, 1.6-11.7; *P* = 0.0005).

**Figure 2:**
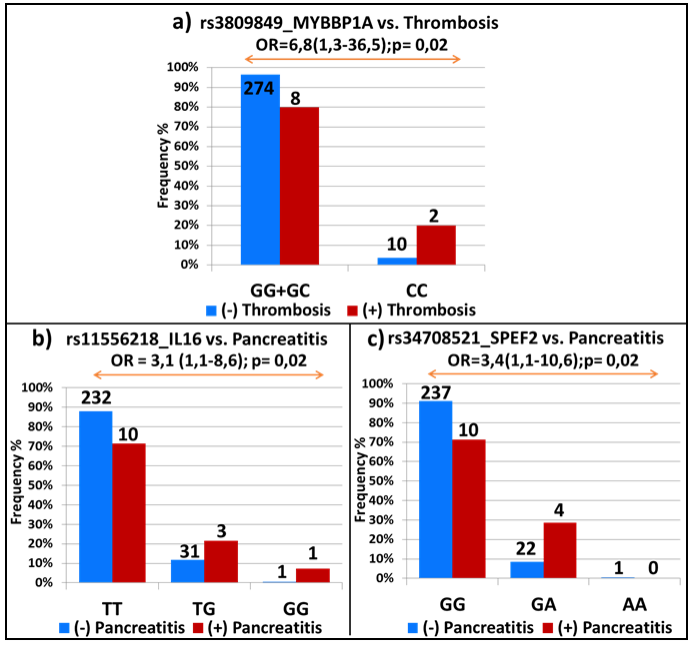
Top-ranking EWAS signals common for several asparaginase-related toxicities SNPs that showed significant associations with one of the asparaginase-related toxicities were further tested for possible associations with the remaining side-effects. Association with thrombosis in **a**. and pancreatitis in **b**. and **c**. The studied association with the OR and 95% CI in brackets is indicated on the top of the graph. The frequency of patients with and without toxicity is represented by red and blue bars, respectively. The number of patients is shown on the top of each bar and the genotypes are indicated at the bottom of the graphs.

In the light of their positive association, each SNP was tested for possible associations with the two other side-effects. Interestingly, on the top of their association with allergy and pancreatitis, homozygote carriers of the variant rs3809849 allele in the *MYBBP1A* gene were associated with a higher risk of thrombosis (OR = 6.8; 95% CI, 1.3-36.5; *p* = 0.02; Figure 2a); whereas, rs11556218 in *IL16* and rs34708521 in *SPEF2* were, in addition to thrombosis, also correlated with pancreatitis (OR = 3.1; 95% CI, 1.1-8.6; *p* = 0.02 and OR = 3.4; 95% CI, 1.1-10.6; *p* = 0.02; Figures 2b and 2c, respectively).

**Figure 3:**
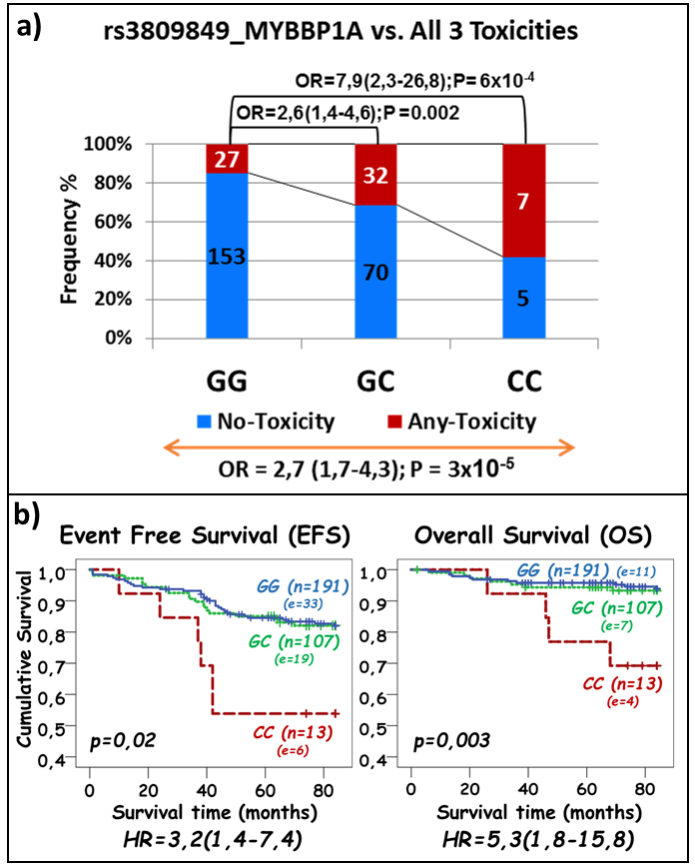
Association of rs3809849 in MYBBP1A gene with ASNase-related toxicities a. and with event free- and overall survival b **a**. The frequency of patients with at least one asparaginase-related toxicity and without any toxicity is represented by the red and blue part of the bar, respectively. The number of samples per category is displayed inside of the bars. The OR with the 95% CI is given when compared to patients with no variant allele (top of the graph) and across all genotype groups (bottom of the graph). **b**. The p-values obtained by the log rank test for the difference across genotypes are provided on each plot. The number of patients represented by each genotype and number of patients with event (in brackets) are indicated next to each curve. Hazard-ratios (HR) obtained through Cox-regression analysis are given with 95% CI.

The risk of any-toxicity increased in additive manner with the minor C allele of the rs3809849 SNP in the *MYBBP1A* gene (OR = 2.7; 95% CI, 1.7-4.3; *p* = 3×10^-5^; Figure 3a). The same SNP was significantly associated with less favorable disease outcomes as homozygous C allele carriers had a reduced EFS (OR = 3.2; 95% CI, 1.4-7.4; *p* = 0.02; Figure 3b) and OS (OR = 5.3; 95% CI, 1.8-15.8; *p* = 0.003; Figure 3b).

In the multivariate analysis, only the association of rs34708521 in *SPEF2* gene with thrombosis lost significance (OR = 4.3; 95% CI, 0.8-22.3; *p* = 0.08), whereas other associations remained significant in their respective models (Supplemental Table S3).

#### Replication analysis

**Figure 5:**
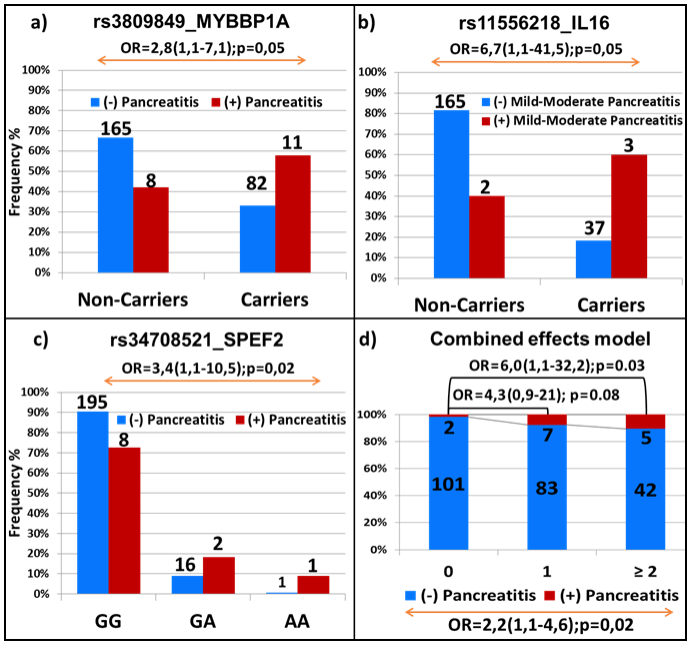
Replication analysis in the independent validation cohort Association of pancreatitis with genetic variations in *MYBBP1A* **a**., *IL16* **b**., *SPEF2* **c**. and in combined effect model **d**. The frequency of patients with and without pancreatitis in **a**., **b**. and **c**. is represented by red and blue bars, respectively. The number and the genotypes are indicated. Combined-effect model in **d**. includes SNPs identified for association with pancreatitis through EWAS of discovery cohort (i.e. rs72755233 in *ADAMTS17*, rs3809849 in *MYBBP1A* and rs9908032 in *SPECC1*). Each bar represents the number of the variant alleles present (i.e. none, one, two or more). The frequency of patients with and without toxicity is represented by the red and blue part of the bar, respectively. The number of samples per category is displayed inside of the bars. The OR with the 95% CI is given when compared to patients with no variants allele (top of the graph) and across groups (bottom of the graph).

Out of the 6 significant associations with allergy and pancreatitis that were confirmed by genotyping in the discovery cohort, the association between rs3809849 in the *MYBBP1A* gene and pancreatitis was replicated in the DFCI cohort (OR = 2.8; 95% CI, 1.1-7.1; *p* = 0.05, Figure 5a). Interestingly, the positive associations that were observed between rs11556218 in *IL16* and rs34708521 in *SPEF2* and the higher risk of pancreatitis were also seen in DFCI cohort (OR = 6.7; 95% CI, 1.1-41.5; *p* = 0.05 in patients with mild and moderate pancreatitis and OR = 3.4; 95% CI, 1.1-10.5; *p* = 0.02, Figures 5b and 5c, respectively). More significant associations were noted for rs3809849 and rs34708521 when analyses were performed in the cohort combining discovery and replication set (*p* = 0.0003 and *p* = 0.008, respectively, Supplemental Table S4). The significant associations with allergies were not replicated, whereas those with thrombosis were not tested since the data were not available in the validation group.

#### Combined effect model

**Figure 4:**
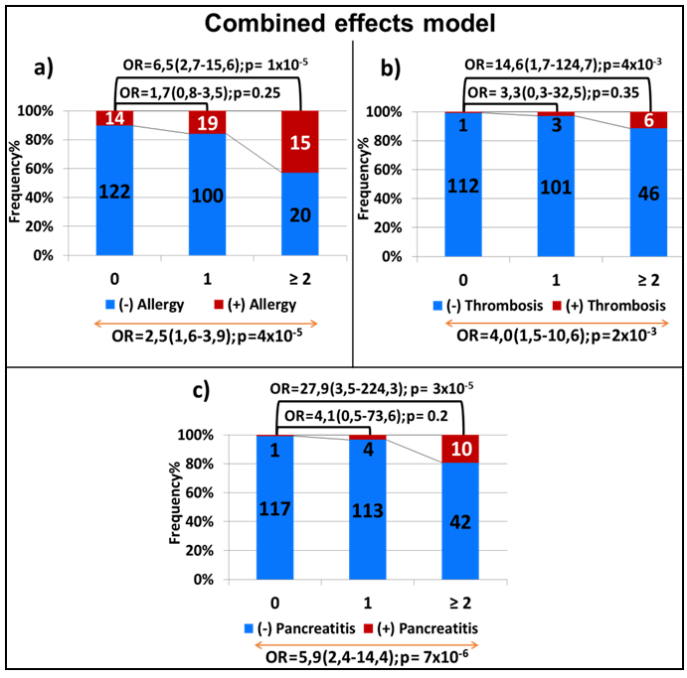
Combined-effect model of the variants associated with allergy a., thrombosis b. and pancreatitis c Each bar represents the number of the variant alleles (i.e. none, one, two or more). The frequency of patients with and without toxicity is represented by the red and blue part of the bar, respectively. The number of samples per category is displayed inside of the bars. The OR with the 95% CI is given when compared to patients with no variants allele (top of the graph) and across genotype groups with increasing number of minor alleles (bottom of the graph).

We next investigated the combined effect of the top-ranked SNPs in each of the toxicities. In this model, a significant correlation was observed between the number of variant alleles carried and the increase in the risk of each of the toxicities. For allergy, the risk associated with an additive effect was 2.5 (95% CI, 1.6-3.9; *p* = 4×10^-5^, Figure 4a), whereas the presence of 2 or more variant alleles was associated with a 6.5-fold increase in the risk of experiencing allergic reactions as compared to not carrying any variant allele (OR = 6.5; 95% CI, 2.7-15.6; *p* = 1×10^-5^, Figure 4a). Similar effect was noted for thrombosis (OR for additive effect = 4.0; 95% CI, 1.5-10.6; *p* = 0.002, Figure 4b). As for pancreatitis, the addition of all 3 variants in the model increased the risk 6-fold (OR = 5.9; 95% CI, 2.4-14.4; *p* = 7×10^-6^, Figure 4c) with carriers of at least two variant alleles being almost 28 times more at risk as compared to those without any variant allele (OR = 27,9; 95% CI, 3,5-224,3; *p* = 3×10^-5^, Figure 4c).

In an attempt to increase the discrimination ability of the model, rs11556218 in *IL16* and rs34708521 in *SPEF2* that were initially investigated for their association with thrombosis but later found to be also associated with pancreatitis, were added to the analysis. In this new comprehensive model with five variants, the groups of 0, 1, 2 and 3 or more variant alleles were compared. The association between the number of minor alleles and the increase in the risk of pancreatitis was directly proportional (OR = 5; 95% CI, 2.4-10.2; *P* = 5×10^-7^, Supplemental Figure S1).

The model combining the 3 SNPs associated with pancreatitis (i.e. rs72755233 in *ADAMTS17*, rs3809849 in *MYBBP1A* and rs9908032 in *SPECC1*) was also replicated in the validation cohort (OR = 2.2; 95% CI, 1.1-4.6; *P* = 0.02, Figure 5d), as also was the comprehensive model with the five variants (OR = 2.6; 95% CI, 1.3-5.4; *P* = 0.005, Supplemental Figure S1). The association was further potentiated in the combined cohort (*p* = 2×10^-6^ and *p* = 1×10^-8^ for the models containing 3 and 5 SNPs, respectively; Supplemental Figure S2).

#### Risk prediction

To assess the performance of the comprehensive combined-effect model in predicting the risk of ASNase-induced pancreatitis, we used the weighted genetic risk score (wGRS) method. [13] A risk score was assigned to each patient by taking the sum of the weighted score of each risk allele across the 5 loci. We then applied these values derived from the discovery cohort to assign the risk scores to patients in the validation cohort. The performance of the model in the discovery, replication and combined cohorts, is summarized in Table 3. The discriminatory ability of the model is reflected by the area under the ROC curve derived from the wGRS. The best sensitivity/specificity values were derived from the OR values greater than 11 corresponding to at least two associated SNPs. The model was successfully validated in the replication and combined cohorts.

**Table 3:** Performance of the comprehensive genetic model in predicting the risk of pancreatitis

Cohort	AUC ± SD.	95% CI	*P*	Sensitivity	Specificty
QcALL	0,80 ± 0,062	68,1 ~ 92,6	1×10^-4^	71%	81%
DFCI	0,78 ± 0,076	63,0 ~ 92,9	3×10^-3^	70%	77%
Combined	0,80 ± 0,049	70,1 ~ 89,1	1×10^-6^	71%	79%

The data were extracted from the receiver operator characteristic (ROC) curves of the comprehensive model for pancreatitis which combines the 5 SNPs associated with this toxicity. The curves were produced by plotting the sensitivity against (1-specificty) of the model using weighted genetic risk scores to estimate the area under the curve in each cohort. The sensitivity and specificity reported in this table are based on an odds ratio greater than 11 for the risk of developing pancreatitis.

AUC, Area Under the Curve; SD, standard deviation; QcALL, Quebec Childhood ALL cohort; DFCI, Dana-Farber Cancer Institute ALL Consortium cohort.

**Figure 6:**
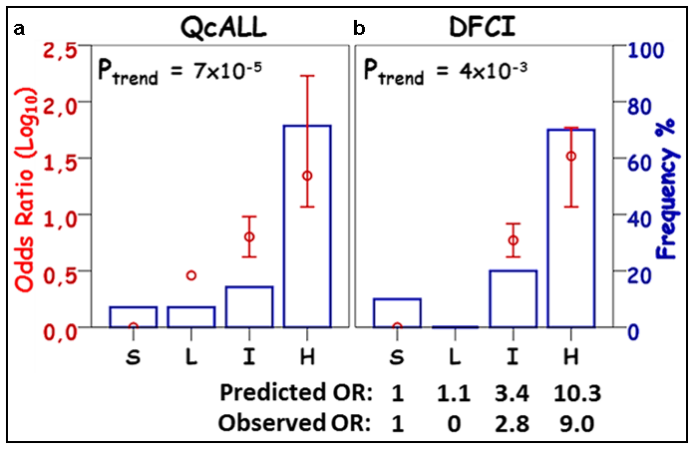
Distribution of patients with pancreatitis among risk groups established using wGRS from the comprehensive combined-effect model in a) QcALL, b) DFCI cohort Risk groups (S, standard; L, low; I, intermediate and H, high) represent the categorical distribution of weighted genetic risk scores (wGRS) of the Comprehensive Combined-effect model containing the 5 SNP associated with pancreatitis in this study (i.e. rs72755233 in *ADAMTS17*, rs3809849 in *MYBBP1A*, rs9908032 in *SPECC1*, rs11556218 in *IL16* and rs34708521 in *SPEF2)*. The wGRS values in **a**. were calculated from the discovery cohort and were used to predict the odds ratios in the validation cohort **b**. The frequency of patients with pancreatitis in each risk group is displayed as a blue lined histogram reflecting the percentage out of the total number of cases. Log(OR) for pancreatitis susceptibility for each risk group (red circle) with a 95% confidence interval and the p-value for the trend across the groups are provided. The groups correspond to the following OR cut-off values: S (1); L ( > 1); I ( > 3.4) and Q4 ( > 10.3) as predicted from the QcALL cohort. The observed ORs per risk group in the DFCI cohort are also provided.

In order to evaluate the efficiency and reproducibility of the model in assigning patients to risk categories, the patients were divided into 4 groups based on the weighted genetic risk scores. Patients who had a score of 0 (indicating the absence of any risk allele) were considered the standard risk category, whereas those who had higher scores were divided into 3 equal groups corresponding to low, intermediate and high risk based on their individually assigned cumulative OR. Distribution of the patients with pancreatitis was compared across the groups and between the two cohorts. The distribution of patients with pancreatitis in the replication cohort (which was based on the predicted ORs) was similar to that of patients from the discovery cohort (who were classified according to their observed ORs), Figure 6. Patients predicted to have the highest risk of pancreatitis (thus assigned to group H) had substantially higher frequency of individuals who actually developed pancreatitis and the observed OR of this group was significantly greater than that of the standard risk group (Figure 6).

## DISCUSSION

Using WES data we identified common genetic variants significantly associated with asparaginase-related side-effects. The rs3809849 in the *MYBBP1A* gene was associated both with allergy and pancreatitis; the significant association with pancreatitis was replicated in the validation cohort. The same SNP was also associated with thrombosis as well as reduction in EFS and OS in discovery cohort. The observed association with EFS and OS could be the result of treatment interruption due to the development of side-effects or could be mediated by ASNase deactivation in the case of allergic reactions. In either situation, the patients would consequently receive a lower ASNase dose intensity, which has been previously shown to be associated with less favourable outcome. [2, 4, 6] Another possible hypothesis involves an increased clearance of dexamethasone driven by anti-asparaginase antibodies which ultimately reduces the overall exposure to this drug and is associated with higher risk of relapse. [14] The effect of other confounding factors such as, for example, leukemia specific mutations, cannot be however ruled out.

*MYBBP1A* gene encodes MYB Binding Protein 1a which is important for early embryonic development as well as many other cellular processes including mitosis, cell cycle control, response to nuclear stress, synthesis of ribosomal DNA and tumoral suppression *via* modulation of the p53 activity. [15, 16] *MYBBP1A* also acts as a co-repressor of the nuclear factor kappaB (NF-kB), [17, 18] a transcription factor activated in response to inflammatory and stress signals, apoptosis and cellular proliferation. Interestingly, a key role of NF-kB in the development of acute pancreatitis has been recently documented. [19] To our knowledge, this is the first study demonstrating an association between *MYBBP1A* gene and the risk of pancreatitis. In general, rs3809849 in *MYBBP1A* gene was rarely investigated. There is only one study which found significant association of this SNP with higher risk of tuberculosis. [18]

Another interesting observation is that 2 loci that were initially investigated for their possible association with thrombosis also showed significant and reproducible associations with pancreatitis. Accordingly, G allele carriers of the rs11556218 SNP in the *IL16* gene and carriers of the A allele in the rs34708521 SNP of the *SPEF2* gene, were at higher risk of pancreatitis in both discovery and replication cohorts. The association with *IL16* is of particular interest because *IL16* gene codes for interleukin-16, a multifactorial cytokine involved in inflammatory and autoimmune diseases as well as cancer risk. [20] In the past few years, rs11556218 has been found to be associated with a wide range of conditions such as endometriosis, [21] Alzheimer's Disease, [22] emphysema, [23] coronary artery disease, [24] ischemic stroke, [25] systemic lupus erythematous, [26] chronic hepatitis B infection, [27] osteoarthritis, [20] overall cancer risk as well as particular cancer types. [28] *SPEF2* stands for “Sperm Flagellar 2” gene which encodes for a protein that is required for correct axoneme development. [29] Even though the association of this gene with thrombosis and pancreatitis might seem counterintuitive, we are tempted to speculate that this might be mediated by the role this gene has in protein dimerization activity and the fact that the protein it encodes is significantly overexpressed in platelets. [30] This finding should be investigated in future studies.

Our analysis also suggests that synergistic interactions might exist between the SNPs identified in each of the studied toxicities, which could explain the markedly significant associations and high odd-ratios in the combined SNPs models. Same combined effect was noted for pancreatitis in the replication set. When all associated SNPs were regarded together, either in combined or comprehensive model, they could explain almost all cases of pancreatitis in both patients’ groups. This was further supported by the model based on wGRS that displayed the best discrimination ability between individuals with and without pancreatitis as confidence limits were substantially above random prediction. Importantly, similar sensitivity and specificity values were observed in the discovery and replication cohorts at odds ratio greater than the chosen threshold which reflects the stability of the model. Furthermore, the prediction model using wGRS values derived from the discovery cohort to assign patients of the validation cohort into risk groups was able to detect far more patients at risk of pancreatitis than any of the SNPs considered alone. In fact, the group of patients predicted to have the highest risk based on their calculated wGRS had a substantial overrepresentation of individuals with pancreatitis compared to all other groups and a significantly higher OR compared to the standard risk group.

This indicates that it would be important to further investigate the utility of using sets of SNPs, rather than individual variants. This EWAS added novel genetic markers to the existing pool of pharmacogenetics modifiers of ASNase treatment that were previously described by several groups including ours, using GWAS and candidate-gene studies (ex. *ATF5* and EFS, [31] ASNS and allergy/pancreatitis, [2] *GRIA1* and hypersensitivity, [32] HLA-DRB1*0701 and allergy, [33] *CPA2* and pancreatitis [8]). Collectively, this rapidly growing pool of markers might become more efficient in explaining the observed inter-individual variability in morbidities associated with anti-leukemia treatment which can eventually help developing genotype guided interventions for patients predisposed to such toxicities. [34]

As per the impact of the sources of ASNase used, the results did not differ significantly when samples of patients who received *Erwinia*-derived ASNase were excluded from the analysis. The only noteworthy observation was related to the association of *IL16* with pancreatitis. On the top of the association with mild-moderate pancreatitis shown earlier in replication cohort (when both ASNase formulations were confounded), *IL16* SNP also showed a significant association with overall pancreatitis in the group treated only with *E. coli* derived formulation in the replication cohort. This difference can be due to the fact that patients treated with *E. coli* ASNase usually have higher rates of ASNase related toxicities. [1, 2] Likewise, the addition of other factors (age, sex, protocol, risk groups) in multivariate model did not affect the results since all of the presented associations remained significant in the multivariate analysis, with the sole exception of rs34708521 in *SPEF2* gene with thrombosis.

There are several limitations to our study. The analyses were done retrospectively as clinical data were inferred from the patients’ medical charts. The distribution of treatment protocols and risk groups varied significantly between the cohorts, which could have introduced variability as patients might have received different ASNase doses. The sample size of the discovery cohort was relatively small and the selected FDR threshold of < 20% was relaxed, which might have increased the number of false-positives, possibly reflected in the high number of EWAS hits. However, the fact that several associations were successfully reproducible in the independent validation cohort supports the validity of the findings. Furthermore, the analysis in the context of a larger sample size provided by the combined cohort further supports the correlation between the SNPs in *MYBBP1A*, *IL16* and *SPEF2* with pancreatitis as the associations gained more significance in the pooled sample. Finally, this study aimed primarily to identify genetic markers that put the patients at risk of developing treatment-related toxicities commonly associated with the use of asparaginase; however, the treatment included other chemotherapeutic agents which makes it difficult to estimate the magnitude of the interaction between asparaginase alone and the genetic composition, requiring experiments in cell lines and animal models to further support the observations.

In conclusion, using WES data in the context of association study was a successful “hypothesis-free” strategy which allowed identifying significant genetic associations with asparaginase-related toxicities in children treated for ALL. Results for pancreatitis were replicated in the independent validation cohort. Even though interesting associations with thrombosis were observed, no replication studies were done due to logistic limitations. Thus, it would be valuable replicating further those results.

## PATIENTS AND METHODS

### Study population and endpoints in the analysis

Discovery cohort consisted of 302 children of European descent from the well-established Quebec Childhood ALL (QcALL) cohort who were diagnosed with childhood ALL at the Sainte-Justine University Hospital Centre (SJUHC), Montreal, QC, Canada, between January 1989 and July 2005. ALL patients received ASNase as part of the Dana-Farber Cancer Institute ALL Consortium protocols DFCI 87-01, 91-01, 95-01, or 00-01 (Table 1). [2, 6, 31, 35] In 95-01 and 00-01, one dose of asparaginase was administered during remission induction, and in all protocols it was administered for 20-30 consecutive weeks during consolidation phase. Details about asparaginase doses and formulation are provided elsewhere. [31, 35] Retrospective review of the medical files was conducted to obtain information on ASNase-related toxicity. Hypersensitivity reactions were defined as adverse local or general manifestations from exposure to asparaginase (flushing, erythema, rash, urticaria, drug fever, dyspnoea, symptomatic bronchospasm, oedema or angio-oedema). [2] Pancreatitis was identified according to the diagnostic criteria of the institution and the guidelines of respective protocols which involved pancreatic enzyme elevation of higher than 3-fold the normal levels along with other clinical signs and symptoms that confirm the diagnosis. [2, 36] Thrombosis was determined by clinical symptoms and confirmed by radiologic imaging based on institutional guidelines. [2, 37]

The replication cohort consisted of 282 children who share similar characteristics with the discovery cohort and who were treated according to the 95-01 and 00-01 protocols. All participants had been previously recruited at one of the nine remaining Dana Farber consortium institutions (i.e. DFCI cohort excluding the SJUHC patients). Information on ASNase related allergy and pancreatitis were available for these patients. Clinical characteristics of both the discovery and replication cohorts are shown in Table 1.

Written informed consent was obtained in accordance with the Declaration of Helsinki from all participants and/or their parents or legal guardians. Institution ethics committees approved the study.

### Whole exome sequencing (WES)

DNA was extracted from peripheral blood or bone marrow samples obtained after remission from 224 childhood ALL patients (QcALL cohort) [38] using standard protocols as described previously. [39] Whole exomes were captured in solution with Agilent's SureSelect Human All Exon 50Mb kits, and sequenced on the Life Technologies SOLiD System (patients mean coverage ~35X). Reads were aligned to the hg19 reference genome using SOLiD LifeScope software. PCR duplicates were removed using Picard. [40] Base quality score recalibration was performed using the Genome Analysis ToolKit (GATK) [41] and QC Failure reads were removed. Cleaned BAM files were used to create pileup files using SAMtool. [42]

Germline variants have been called using SNooPer [43] a variant caller based on a machine learning algorithm that uses a subset of variant positions from the sequencing output for which the class is known, either actual variation or sequencing error, to train a data-specific model.

The annotation of the identified germline variants was performed using ANNOVAR. [44] Only missense, nonsense and variations in splicing sites were conserved. The predicted effect of missense variants on the protein function was assessed *in silico* using Sift (≤0.05) [45] and Polyphen2 (≥0.5). [46] Minor allele frequencies higher than 5% were derived from the 1000 Genomes (European population) [47] and the NHLBI GO Exome Sequencing Project (European population, ESP). [48]

Fisher's Exact test (allelic association) and Cochran-Armitage trend test, implemented in PLINK [49], were used for an association study. Adjustment for multiple testing was performed by bootstrap false discovery rate (FDR) [50] method; the SNPs retained for further analysis had FDR lower than 20%.

### Validation of top-ranking EWAS signals by genotyping

Genotyping of top ranking EWAS signals was either performed at the McGill University and Génome Québec Innovation Centre through Sequenom genotyping platform or by allele-specific oligonucleotides (ASOs) hybridization as described earlier. [51] Comparison between genotypes and ASNase related complication was performed for each of the SNPs by χ^2^ test or Fisher test. For significant associations, the genetic model that was most representative of the effect of the variant (i.e. additive, dominant, or recessive) was tested as well. The genotype-associated risk was expressed as odds ratio (OR) with 95% confidence interval (CI). Survival differences in terms of event-free-survival (EFS) and overall survival (OS) were estimated using Kaplan-Meier analysis for patients with different genotypes and were assessed using log-rank test. Patients were followed for up to five years after the last therapeutic dose and an event was defined as induction failure, relapse, second malignancy or death from any cause. Combined effect of associated SNPs was tested by recoding genotypes as having none, one or two and more alleles at risk. Logistic regression was used for multivariate analysis which included beside genotypes: sex, age ( < 10 years or ≥ 10 years), risk (standard or high), DFCI protocol and asparaginase formulation (*E.coli* or *Ervinia*) as categorical variables. Statistical analyses were performed with IBM SPSS Statistics for Windows, Version 22.0. (IBM Corp. Armonk, NY).

### Risk prediction

Weighted genetic risk score (wGRS) method was used to predict the risk of developing ASNase induced pancreatitis based on the cumulative combined effect of all SNPs found to be associated with this toxicity in the current study. The wGRS was estimated from the number of risk alleles by calculating the sum of weighted ln(OR) for each allele as explained elsewhere. [13] The performance of the comprehensive model in classifying patients based on their individual wGRS was assessed by calculating the area under the receiver operator characteristic (ROC) curves.

## SUPPLEMENTARY MATERIALS FIGURES AND TABLES





## Abbreviations

ALL: Acute Lymphoblastic Leukemia
ASN: Asparagine
ASNase: Asparaginase
AUC: Area Under the Curve
CI: Confidence Interval
DFCI: Dana-Farber Cancer Institute
*E.coli*: *Escherichia coli*
EFS: Event-free Survival
*Erwinia*: *Erwinia chrysanthemi*
EWAS: Exome-Wide Association Study
FDR: False Discovery Rate
HR: Hazard Ratio
NF-kB: Nuclear Factor kappaB
OR: Odds Ratio
OS: Overall Survival
PCR: Polymerase Chain Reaction
QcALL: Quebec Childhood Acute Lymphoblastic Leukemia
ROC: Receiver Operator Characteristic
SJUHC: Sainte-Justine University Hospital Centre
SNP: Single Nucleotide Polymorphism
WES: Whole Exome Sequencing
wGRS: weighted Genetic Risk Score
