# Supplementary material for: Whole-exome sequencing identified genetic risk factors for asparaginase-related complications in childhood ALL patients
Source: Oncotarget. 2017 May 17;8(27):43752–67. doi: 10.18632/oncotarget.17959 (PMC5546438; doi:10.18632/oncotarget.17959)
Supplement: Supplementary file 1 [file oncotarget-08-43752-s001.pdf]

Whole-exome sequencing identified genetic risk factors for asparaginase-related complications in childhood ALL patients

Supplementary Material

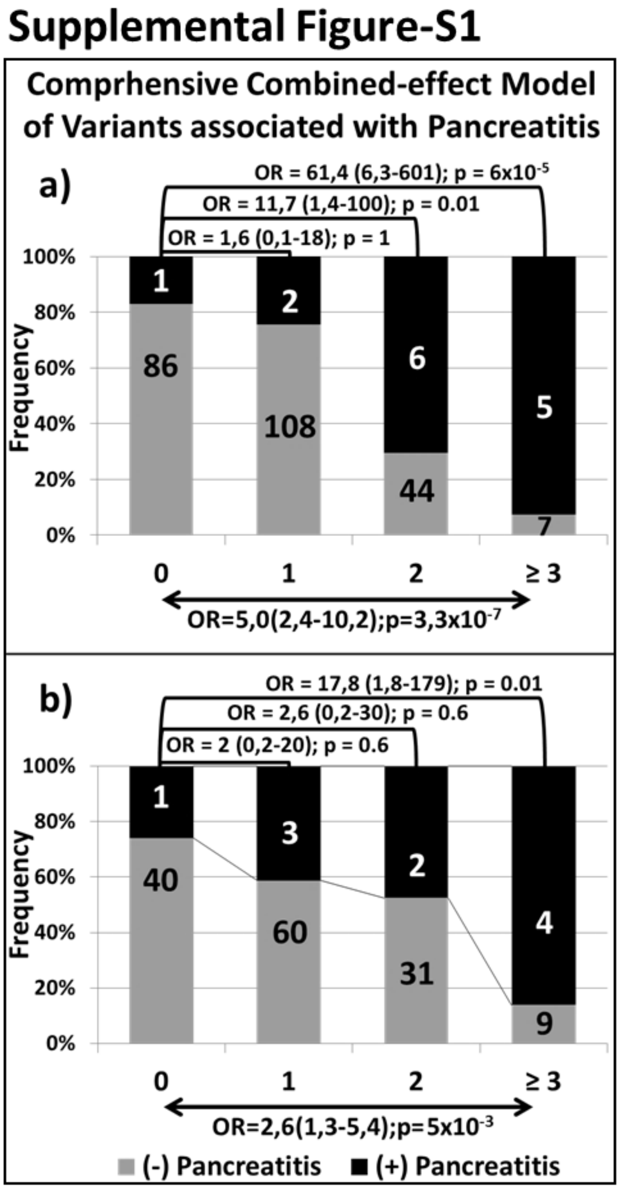

Supplemental Figure S1: Comprehensive combined-effect model of all SNPs significantly associated with pancreatitis The combined effect of all five SNPs found to have significant associations with pancreatitis throughout the study (i.e. rs72755233 in *ADAMTS17*, rs3809849 in *MYBBP1A,* rs9908032 in *SPECC1,* rs11556218 in *IL16* and rs34708521 in *SPEF2*) was investigated in both a) the discovery cohort and b) the replication cohort. The groups of 0, 1, 2 and 3 or more variant alleles were compared. The association between the number of minor alleles and the increase in the risk of pancreatitis was directly proportional (OR and 95%CI at the bottom of the graph). Each bar represents the number of the variant alleles (i.e. none, one, two, three or more). The frequency of patients with and without pancreatitis is represented by the black and grey part of the bar. The number of samples per category is displayed inside of the bar. Carriers of 3 or more variant alleles were associated with a significant increase in the risk of pancreatitis (OR and 95% CI at the top of the graph).

Supplemental Figure-S2

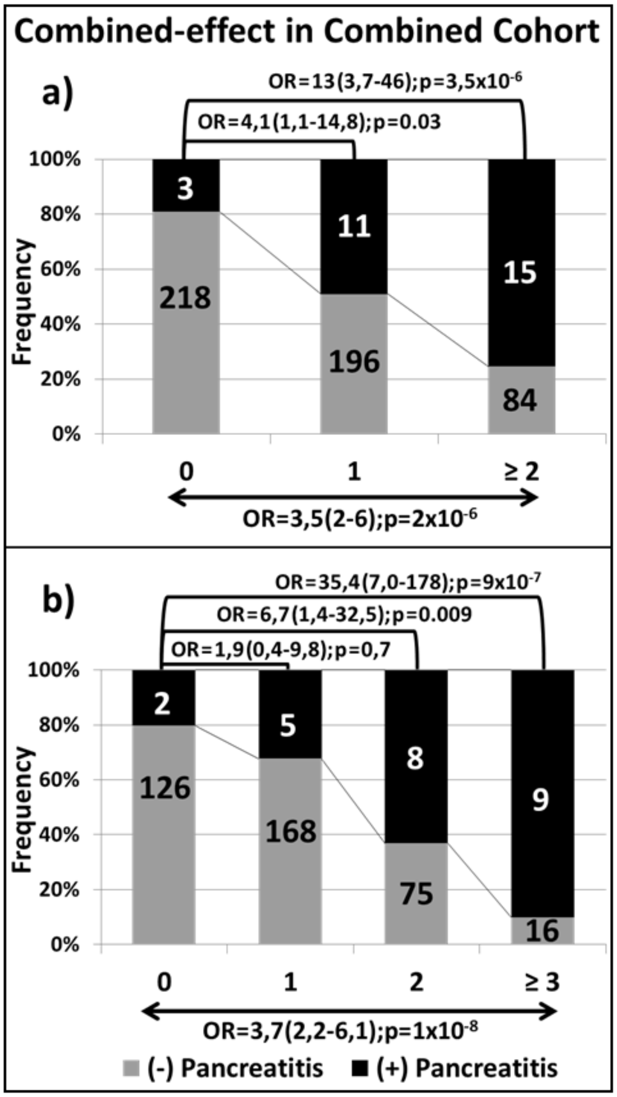

Supplemental Figure S2: Combined-effect analysis in Combined-cohort for the a) 3 SNPs and b) 5 SNPs significantly associated with pancreatitis. The combined effect of SNPs associated with pancreatitis was analysed in the combined cohort both for a) the model containing only the three SNPs significantly associated with pancreatitis in the EWAS (i.e. rs72755233 in *ADAMTS17*, rs3809849 in *MYBBP1A* and rs9908032 in *SPECC1*) and b) the comprehensive model additionally containing rs11556218 in *IL16* and rs34708521 in *SPEF2*. The patients were assigned to groups based on the number of risk alleles indicated at the bottom of each bar. The association between the number of minor alleles and the increase in the risk of pancreatitis was directly proportional (OR and 95%CI at the bottom of the graph). The frequency of patients with and without pancreatitis is represented by the black and grey part of the bar. The number of samples per category is displayed inside of the bar. The OR with 95% CI for each risk group (as compared to the group of patients not having any of the risk alleles) is displayed at the top of the graph.

For Supplementary Tables see in supplementary Files
