# Supplementary material for: Whole-exome sequencing identified genetic risk factors for asparaginase-related complications in childhood ALL patients
Source: Oncotarget. 2017 May 17;8(27):43752–67. doi: 10.18632/oncotarget.17959 (PMC5546438; doi:10.18632/oncotarget.17959)
Supplement: Supplementary file 2 [file oncotarget-08-43752-s002.docx]

**Supplemental Table S1. Association of WES data with asparaginase-related complications**

| **Gene_SNP** | **Toxicity** | **MinorAlleleFrequency (%)** | **P-value Allelic Association (Sequencing Data)** | **FDR** | **P-value Association by Genotype (Sequencing Data)** | **P-value Association by Genotype (Genotyping Data)*** |
| --- | --- | --- | --- | --- | --- | --- |
| KIAA1107_rs565156: C > T | Allergy | 1% | 1,23E-05 | 1,4% | 2,00E-03 | - |
| MAP3K1_rs702689: G > C | Allergy | 1% | 1,23E-05 | 1,4% | 2,00E-03 | - |
| CPNE1_rs12481228: G > C | Allergy | 2% | 1,96E-05 | 1,5% | 8,31E-04 | - |
| **SLC7A13_rs9656982: A > G** | **Allergy** | **9%** | **1,34E-04** | **7,4%** | **9,91E-04** | ***1,63E-02*** |
| KCNJ15_rs2230033: G > A | Allergy | 50% | 3,57E-04 | 8,9% | 1,81E-03 | - |
| OTOF_rs4665855: G > A | Allergy | 32% | 5,18E-04 | 8,9% | 1,53E-03 | - |
| **HLA-DPA1_rs199711661: T > C** | **Allergy** | **5%** | **8,57E-04** | **8,9%** | **3,42E-03** | 1,58E-01 |
| CETP_rs5880: G > C | Allergy | 3% | 1,01E-03 | 8,9% | 8,00E-04 | - |
| **PRR15_rs112093295: C > A** | **Allergy** | **6%** | **1,25E-03** | **8,9%** | **3,66E-03** | 1,29E-01 |
| ZNF880_rs14048: G > A | Allergy | 2% | 1,43E-03 | 8,9% | 1,30E-03 | - |
| GTPBP5_rs6062133: G > A | Allergy | 2% | 1,50E-03 | 8,9% | 1,40E-03 | - |
| ALOXE3_rs3027229: G > C | Allergy | 6% | 1,61E-03 | 8,9% | 1,33E-03 | - |
| PKD2L2_rs1880458: G > A | Allergy | 1% | 1,92E-03 | 8,9% | 3,88E-02 | - |
| PCDHA7_rs61730623: G > A | Allergy | 1% | 2,31E-03 | 9,8% | 2,20E-03 | - |
| OR2M7_rs7555310: A > G | Allergy | 1% | 2,40E-03 | 9,8% | 2,30E-03 | - |
| LILRB3_rs61734493: C > A | Allergy | 1% | 2,40E-03 | 9,8% | 2,30E-03 | - |
| ARAP3_rs1031904: C > G | Allergy | 2% | 2,42E-03 | 9,8% | 1,78E-02 | - |
| SLC22A25_rs11231397: C > G | Allergy | 8% | 2,51E-03 | 9,9% | 1,38E-02 | - |
| FLG_rs12405278: G > A | Allergy | 3% | 2,55E-03 | 9,9% | 2,20E-03 | - |
| C17orf80_rs745143: T > C | Allergy | 50% | 2,88E-03 | 10,9% | 7,69E-03 | - |
| KRT72_rs11170183: C > A | Allergy | 4% | 3,29E-03 | 12,1% | 2,47E-02 | - |
| **LILRB2_rs386056: C > T** | **Allergy** | **7%** | **3,47E-03** | **12,6%** | **3,13E-03** | 1,62E-01 |
| NACAD_rs61740895: G > A | Allergy | 21% | 3,91E-03 | 14,0% | 2,40E-02 | - |
| RP1L1_rs4841399: G > C | Allergy | 4% | 5,10E-03 | 18,0% | 3,84E-02 | - |
| ACSM2A_rs1133607: C > T | Allergy | 3% | 5,55E-03 | 19,0% | 4,70E-03 | - |
| **MYBBP1A_rs3809849: G > C** | **Allergy** | **11%** | **5,60E-03** | **19,0%** | **2,07E-03** | ***2,34E-03*** |
| CD6_rs11230563: C > T | Allergy | 2% | 5,66E-03 | 19,0% | 3,82E-02 | - |
| FBXL6_rs61746974: C > G | Allergy | 3% | 6,07E-03 | 19,8% | 2,19E-02 | - |
| DYNC2H1_rs17301182: C > T | Allergy | 7% | 6,07E-03 | 19,8% | 1,09E-02 | - |
| OR5D18_rs297081: A > G | Allergy | 2% | 1,24E-06 | 0,2% | 6,00E-04 | - |
| KCNMB3_rs7645550: C > T | Allergy | 14% | 1,20E-04 | 7,3% | 1,10E-03 | - |
| COL9A3_rs61734651: C > T | Allergy | 4% | 1,55E-04 | 7,3% | 1,21E-03 | - |
| SNX15_rs495820: C > T | Allergy | 2% | 1,88E-04 | 7,3% | 9,14E-03 | - |
| CFB_rs4151667: T > A | Allergy | 2% | 2,42E-04 | 7,3% | 5,83E-03 | - |
| **FBXO24_rs11768465: C > T** | **Allergy** | **11%** | **2,92E-04** | **7,3%** | **1,25E-03** | 6,06E-01 |
| TTC3_rs61999340: C > G | Allergy | 6% | 3,23E-04 | 7,3% | 2,00E-04 | - |
| **YTHDC2_rs75714066: G > C** | **Allergy** | **5%** | **3,51E-04** | **7,3%** | **2,00E-04** | ***9,63E-03*** |
| OR52J3_rs58664826: G > A | Allergy | 7% | 3,89E-04 | 7,3% | 1,36E-03 | - |
| **SENP6_rs17414086: C > T** | **Allergy** | **14%** | **6,00E-04** | **7,8%** | **1,26E-03** | 6,98E-01 |
| KIAA1551_rs3759302: T > A | Allergy | 8% | 7,54E-04 | 9,1% | 7,83E-03 | - |
| NPHS1_rs3814995: C > T | Allergy | 5% | 1,33E-03 | 15,0% | 1,67E-02 | - |
| FERMT1_rs62200482: G > A | Allergy | 7% | 1,43E-03 | 15,1% | 1,17E-02 | - |
| **P2RY11,PPAN-P2RY11_rs3745601: G > A** | Allergy | 2% | 1,92E-03 | 19,1% | 2,13E-02 | - |
| **PKD2L1_rs6584356: C > A** | **Thrombosis** | **6%** | **1,47E-09** | **0,0%** | **6,92E-12** | ***2,88E-07*** |
| MYO15A_rs712270: A > T | Thrombosis | 2% | 1,51E-09 | 0,0% | 2,08E-09 | - |
| PKD1L2_rs7185774: C > T | Thrombosis | 11% | 2,10E-06 | 0,0% | 4,45E-07 | - |
| **RIN3_rs3742717: C > T** | **Thrombosis** | **5%** | **1,87E-05** | **0,1%** | **8,00E-06** | ***1,02E-03*** |
| C2orf61_rs815804: G > T | Thrombosis | 14% | 1,95E-05 | 0,1% | 3,85E-07 | - |
| MYH7B_rs3746435: G > C | Thrombosis | 4% | 5,51E-05 | 0,3% | 4,38E-05 | - |
| CCDC135_rs3809611: C > T | Thrombosis | 21% | 1,36E-04 | 0,6% | 3,18E-04 | - |
| DHX37_rs11057939: C > T | Thrombosis | 12% | 2,32E-04 | 1,0% | 1,07E-03 | - |
| NRN1L_rs73594554: G > A | Thrombosis | 9% | 3,49E-04 | 1,5% | 1,10E-06 | - |
| **SPEF2_rs34708521: G > A** | **Thrombosis** | **5%** | **4,04E-04** | **1,6%** | **3,00E-04** | ***2,30E-02*** |
| **ESYT2_rs2305475: A > G** | **Thrombosis** | **5%** | **4,04E-04** | **1,6%** | **5,03E-04** | 4,39E-01 |
| POU6F2_rs2074936: C > T | Thrombosis | 5% | 4,89E-04 | 1,9% | 1,06E-04 | - |
| EPPK1_rs11781942: G > A | Thrombosis | 14% | 7,45E-04 | 2,7% | 1,79E-08 | - |
| NUP153_rs61744976: G > C | Thrombosis | 16% | 2,26E-03 | 6,7% | 6,60E-03 | - |
| CCDC41_rs74340001: G > A | Thrombosis | 7% | 2,66E-03 | 7,6% | 1,16E-04 | - |
| **TLR3_rs3775291: C > T** | **Thrombosis** | **28%** | **2,76E-03** | **7,7%** | **1,95E-03** | 7,93E-02 |
| PRR16_rs17853861: C > A | Thrombosis | 16% | 2,76E-03 | 7,7% | 5,30E-04 | - |
| FAM26F_rs11544160: G > A | Thrombosis | 7% | 3,41E-03 | 9,3% | 2,30E-03 | - |
| SFI1_rs16989291: T > C | Thrombosis | 7% | 3,81E-03 | 9,7% | 2,99E-03 | - |
| SSC5D_rs925878: C > T | Thrombosis | 11% | 3,86E-03 | 9,7% | 1,53E-02 | - |
| **SLC39A12_rs62619938: C > T** | **Thrombosis** | **7%** | **4,45E-03** | **10,4%** | **4,08E-03** | ***2,27E-03*** |
| TMEM123_rs11547915: C > A | Thrombosis | 7% | 4,45E-03 | 10,4% | 3,10E-03 | - |
| **MPEG1_rs7926933: G > A** | **Thrombosis** | **7%** | **4,45E-03** | **10,4%** | **7,47E-03** | ***7,33E-03*** |
| **IL16_rs11556218: T > G** | **Thrombosis** | **7%** | **4,45E-03** | **10,4%** | **7,47E-03** | ***4,16E-03*** |
| **CSTL1_rs3746737: C > T** | **Thrombosis** | **7%** | **4,45E-03** | **10,4%** | **2,78E-03** | ***1,54E-02*** |
| FAM198A_rs3732858: G > A | Thrombosis | 7% | 5,90E-03 | 12,6% | 5,79E-03 | - |
| LRRN2_rs11588857: G > A | Thrombosis | 12% | 6,04E-03 | 12,6% | 1,27E-06 | - |
| BRD8_rs11750814: G > A | Thrombosis | 12% | 6,37E-03 | 12,9% | 1,70E-03 | - |
| SGCG_rs17314986: G > A | Thrombosis | 13% | 8,59E-03 | 16,4% | 7,00E-03 | - |
| C20orf85_rs17440813: A > G | Thrombosis | 2% | 2,94E-11 | 0,0% | 3,66E-07 | - |
| **PRR5L_rs62621409: A > G** | **Thrombosis** | **5%** | **5,32E-04** | **9,6%** | **1,73E-05** | ***1,18E-02*** |
| F13A1_rs5988: C > G | Thrombosis | 21% | 9,43E-04 | 12,1% | 8,48E-04 | - |
| PKD1L1_rs76100363: G > A | Pancreatitis | 2% | 3,72E-08 | 0,0% | 3,27E-07 | - |
| OR5K3_rs13068323: G > A | Pancreatitis | 10% | 3,19E-05 | 0,5% | 2,22E-04 | - |
| **PARP15_rs12489170: G > A** | **Pancreatitis** | **11%** | **6,32E-05** | **0,9%** | **5,81E-08** | 5,31E-02 |
| **ADAMTS17_rs72755233: G > A** | **Pancreatitis** | **11%** | **1,57E-04** | **1,8%** | **8,27E-05** | ***1,25E-03*** |
| FBXL6_rs61746974: C > G | Pancreatitis | 3% | 1,86E-04 | 2,0% | 1,25E-04 | - |
| ELL3_rs2277531: G > C | Pancreatitis | 6% | 3,53E-04 | 3,6% | 4,32E-04 | - |
| DNAH9_rs3744581: A > G | Pancreatitis | 13% | 4,65E-04 | 3,8% | 3,00E-04 | - |
| **PDZRN4_rs285584: G > A** | **Pancreatitis** | **9%** | **6,21E-04** | **4,1%** | **5,65E-03** | 5,35E-01 |
| SPATA21_rs41269193: G > T | Pancreatitis | 4% | 6,49E-04 | 4,1% | 1,42E-06 | - |
| PREX1_rs41283558: C > G | Pancreatitis | 13% | 7,71E-04 | 4,7% | 1,78E-04 | - |
| **PYCRL_rs2242089: C > T** | **Pancreatitis** | **10%** | **9,73E-04** | **5,8%** | **1,30E-03** | 9,52E-02 |
| CCDC8_rs2279517: C > G | Pancreatitis | 4% | 1,18E-03 | 6,7% | 9,00E-04 | - |
| GSTZ1_rs7975: G > A | Pancreatitis | 4% | 1,29E-03 | 7,1% | 2,44E-03 | - |
| DNHD1_rs4282961: C > A | Pancreatitis | 23% | 1,99E-03 | 10,7% | 2,55E-03 | - |
| **SEPT_4_rs17741424: T > A** | **Pancreatitis** | **11%** | **2,30E-03** | **11,9%** | **1,17E-03** |  |
| AKAP13_rs4075256: T > C | Pancreatitis | 40% | 2,60E-03 | 13,1% | 1,16E-02 | - |
| **TJP2_rs77236826: A > G** | **Pancreatitis** | **8%** | **2,75E-03** | **13,5%** | **9,62E-07** | 2,95E-01 |
| **MYBBP1A_rs3809849: G > C** | **Pancreatitis** | **11%** | **3,47E-03** | **16,1%** | **3,24E-03** | ***2,34E-03*** |
| OR52R1_rs7941731: A > G | Pancreatitis | 35% | 4,13E-03 | 18,5% | 1,69E-02 | - |
| PHLPP2_rs61733127: A > G | Pancreatitis | 16% | 4,20E-03 | 18,5% | 1,44E-03 | - |
| OR4D2_rs74730740: C > T | Pancreatitis | 8% | 4,64E-03 | 19,0% | 9,33E-03 | - |
| DFNB31_rs12339210: G > C | Pancreatitis | 8% | 4,64E-03 | 19,0% | 9,33E-03 | - |
| **MUC16_rs12150888: G > T** | **Pancreatitis** | **25%** | **4,65E-03** | **19,0%** | **1,24E-03** | 4,36E-01 |
| F13A1_rs5987: C > T | Pancreatitis | 4% | 3,35E-05 | 0,7% | 2,20E-05 | - |
| C19orf59_rs72996468: A > G | Pancreatitis | 4% | 4,73E-05 | 0,8% | 1,00E-03 | - |
| MAVS_rs7262903: C > A | Pancreatitis | 16% | 1,11E-04 | 1,4% | 2,16E-04 | - |
| NPSR1_rs7809642: C > T | Pancreatitis | 5% | 3,62E-04 | 2,9% | 4,04E-06 | - |
| **GJB7_rs35259282: C > T** | **Pancreatitis** | **5%** | **4,08E-04** | **2,9%** | **1,86E-06** | 1,87E-01 |
| FCRL6_rs61823162: C > T | Pancreatitis | 9% | 5,66E-04 | 2,9% | 5,91E-04 | - |
| **LRRC31_rs35923425: C > G** | **Pancreatitis** | **6%** | **1,29E-03** | **5,0%** | **1,33E-03** | 2,78E-01 |
| MMP17_rs11835665: G > A | Pancreatitis | 6% | 1,75E-03 | 5,9% | 2,23E-03 | - |
| **RAB3GAP2_rs2289189: C > G** | **Pancreatitis** | **7%** | **3,19E-03** | **9,3%** | **2,20E-03** | 6,00E-02 |
| **SPECC1_rs9908032: C > G** | **Pancreatitis** | **11%** | **3,84E-03** | **10,4%** | **1,37E-06** | ***4,98E-05*** |
| CARD10_rs9610775: C > T | Pancreatitis | 7% | 3,96E-03 | 10,4% | 1,89E-02 | - |
| **UBD_rs2076485: A > G** | **Pancreatitis** | **23%** | **4,42E-03** | **11,0%** | **3,26E-05** | 9,22E-02 |
| PCDH15_rs11004439: A > C | Pancreatitis | 17% | 5,37E-03 | 13,0% | 3,54E-03 | - |
| ERCC6_rs2228527: T > C | Pancreatitis | 24% | 5,84E-03 | 13,6% | 2,15E-02 | - |
| **HRG_rs2228243: A > G** | **Pancreatitis** | **18%** | **5,97E-03** | **13,6%** | **5,74E-03** | 8,95E-01 |
| C3orf20_rs9821143: G > A | Pancreatitis | 31% | 6,39E-03 | 13,6% | 8,97E-03 | - |
| GTF2A1L_rs940389: G > C | Pancreatitis | 33% | 1,18E-02 | 18,4% | 6,81E-04 | - |

The SNPs selected for validation through genotyping are highlighted and those that remained significant are depicted in dark grey color. *p value reflects the difference across genotype groups regardless of genetic model. Further analysis in accordance to appropriate models is presented in Table 2. CSTL1 and PRR5L were not considered further, as association did not follow any genetic model.

**Supplemental Table S2. Function and differential protein expression of genes selected for further investigation.**

| **Gene** | **Toxicity** | **Full Name** | **Function** | **Protein Differential Expression** |
| --- | --- | --- | --- | --- |
| **SLC7A13** | **Allergy** | Solute Carrier Family 7 Member 13 | Amino acid transmembrane transporter activity | Kidney Cortex and Testis |
| **HLA-DPA1** | **Allergy** | Major Histocompatibility Complex, Class II, DP Alpha 1 | Central role in the immune system | B-lymphocyte, Lymph node and Lung |
| **PRR15** | **Allergy** | Proline Rich 15 | May have a role in proliferation and/or differentiation | Plasma, Platelet, Fetal ovary, Heart, and Fetal testis |
| **LILRB2** | **Allergy** | Leukocyte Immunoglobulin Like Receptor B2 | Immunoregulatory interactions between Lymphoid and non-Lymphoids cell and Immune System | Neutrophil, Monocytes and Cervix |
| **MYBBP1A** | **Allergy** | MYB Binding Protein 1a | Role in various cellular processes including response to nucleolar stress, tumor suppression and synthesis of ribosomal DNA | T-lymphocyte, Pancreas, Peripheral blood mononuclear cells and Heart |
| **FBXO24** | **Allergy** | F-Box Protein 24 | Phosphorylation-dependent ubiquitination (ubiquitin-protein transferase activity) | Pancreatic juice, Platelets and Testis |
| **YTHDC2** | **Allergy** | YTH Domain Containing 2 | Nucleic acid binding and helicase activity. Linked to susceptibility to pancreatic cancer in human patients | Pancreatic juice, Ovary and Cerebrospinal fluid |
| **SENP6** | **Allergy** | SUMO1/Sentrin Specific Peptidase 6 | Cysteine-type peptidase activity and SUMO-specific protease activity | CD8 T-cells, Peripheral blood mononuclear cells and Testis |
| **PKD2L1** | **Thrombosis** | Polycystin 2 Like 1 | Calcium-regulated nonselective cation channel | Plasma and Colon |
| **RIN3** | **Thrombosis** | Ras Interaction/Interference Protein 3 | Vesicle-mediated transport, GTPase activator activity and Rab guanyl-nucleotide exchange factor activity | Peripheral blood mononuclear cells, Monocytes, NK cells, B-lymphocyte and Lymph node |
| **SPEF2** | **Thrombosis** | Sperm Flagellar 2 | Protein dimerization activity | Platelet, Fetal Brain and Fetal ovary |
| **ESYT2** | **Thrombosis** | Extended Synaptotagmin 2 | Calcium ion binding and phosphatidylinositol binding and may play a role in cellular lipid transport | Peripheral blood mononuclear cells |
| **TLR3** | **Thrombosis** | Toll Like Receptor 3 | Induces the activation of NF-kappaB and the production of type I interferons. Fundamental role in pathogen recognition and activation of innate immunity | Megakaryocytes, Platelets, Immature Dendritic cells, Pancreas and Nasal epithelium |
| **SLC39A12** | **Thrombosis** | Solute Carrier Family 39 Member 12 | Metal ion transmembrane transporter activity and zinc ion transmembrane transporter activity. Thought to be involved in platelet function | Heart, Retina and Frontal cortex |
| **MPEG1** | **Thrombosis** | Macrophage Expressed 1 | Cell cycle. Pathogen Recognition and Activation of the Innate Immune Response. | Peripheral blood mononuclear cells, Monocytes and Testis |
| **IL16** | **Thrombosis** | Interleukin 16 | Cytokine activity, Chemoattractant, a modulator of T cell activation, and an inhibitor of HIV replication | Lymph node, CD8 T-cells, T-lymphocyte, and Peripheral blood mononuclear cells |
| **CSTL1** | **Thrombosis** | Cystatin Like 1 | Cysteine-type endopeptidase inhibitor activity found in a variety of human fluids and secretions. | Testis |
| **PRR5L** | **Thrombosis** | Proline Rich 5 Like | Ubiquitin protein ligase binding. Related pathways are mTOR signalling and PI3K / Akt Signaling | Spleen, Colon, and Brain |
| **PARP15** | **Pancreatitis** | Poly(ADP-Ribose) Polymerase Family Member 15 | NAD+ ADP-ribosyltransferase activity: transfers ADP-ribose from nicotinamide dinucleotide (NAD) to Glu/Asp residues on the substrate protein | Adipocyte and B-lymphocyte |
| **ADAMTS17** | **Pancreatitis** | A Disintegrin-Like And Metalloprotease (Reprolysin Type) With Thrombospondin Type 1 Motif, 17 | Endopeptidase and Metalloendopeptidase activity | Plasma and Esophagus |
| **PDZRN4** | **Pancreatitis** | PDZ Domain Containing Ring Finger 4 | Ubiquitin-protein transferase activity and ubiquitin protein ligase activity. Potential role as tumor suppressor | Platelet |
| **PYCRL** | **Pancreatitis** | Pyrroline-5-Carboxylate Reductase-Like | Pyrroline-5-carboxylate reductase activity. Involved in Arginine and proline metabolism | Multiple tissues |
| **SEPT_4** | **Pancreatitis** | Septin 4 | GTPase activity. Apoptosis Modulation and Signaling. Localized to the mitochondria, and has a role in apoptosis and cancer. May play a role in cytokinesis and platelet secretion | Frontal cortex, Spinal cord, Retina, and Spleen |
| **TJP2** | **Pancreatitis** | Tight Junction Protein 2 | Blood-Brain Barrier and Immune Cell Transmigration: VCAM-1/CD106 Signaling Pathways | Peripheral blood mononuclear cells, Platelet, Nasal epithelium and Cervix |
| **MYBBP1A** | **Pancreatitis** | MYB Binding Protein 1a | Role in various cellular processes including response to nucleolar stress, tumor suppression and synthesis of ribosomal DNA | Pancreas, T-lymphocyte, Peripheral blood mononuclear cells and Heart |
| **MUC16** | **Pancreatitis** | Mucin-16 | Role in immune system by providing a protective, lubricating barrier against particles and infectious agents at mucosal surfaces. | Platelet, Liver, Cervix and Breast. Expression is significantly increased in Pancreatic Cancer |
| **GJB7** | **Pancreatitis** | Gap Junction Protein Beta 7 | Vesicle-mediated transport and Gap junction trafficking. Contributes to leukemia cell communication and chemosensitivity | Unspecified |
| **LRRC31** | **Pancreatitis** | Leucine Rich Repeat Containing 31 | Unspecified | Thyroid, Tonsil and Esophagus |
| **RAB3GAP2** | **Pancreatitis** | RAB3 GTPase Activating Protein Subunit 2 | Vesicle-mediated transport. Involved in regulated exocytosis of neurotransmitters and hormones | Lymph node |
| **SPECC1** | **Pancreatitis** | Sperm Antigen With Calponin Homology And Coiled-Coil Domains 1 | Is a novel fusion partner to PDGFRB in juvenile myelomonocytic leukemia with t(5;17)(q33;p11.2). | Peripheral blood mononuclear cells, Lung, Testis and some cancer cell lines |
| **UBD** | **Pancreatitis** | Ubiquitin D | Regulates IRE1α/JNK-dependent apoptosis in pancreatic beta cells. Role in regulation of NF-kappa-B signaling | Islet of Langerhans, Monocytes, Liver, Ovary |
| **HRG** | **Pancreatitis** | Histidine Rich Glycoprotein | Binds heme, dyes and divalent metal ions. Involved in inhibition of fibrinolysis and the reduction of inhibition of coagulation. Normalizes tumor vessels and promotes antitumor immunity | Serum, Plasma, Monocytes and Synovial fluid |

The name, function and differential protein expression is provided for each of the genes selected for confirmation by genotyping after the initial filtration of the EWAS signals. The genes were selected based on a biological function or expression profile that could be relevant to the studied toxicity.

**Supplemental Table S3. Multivariate analysis for primary and secondary associations confirmed by genotyping**

| **SNP** | **Toxicity** | **P** | **OR (95%-CI)** |
| --- | --- | --- | --- |
| **SLC7A13_rs9656982: A > G** | Allergy | 0,03 | 2,1 (1,1-4,0)^a^ |
| **MYBBP1A_rs3809849: G > C** | Allergy | 0,002 | 2,3 (1,4-3,9)^a^ |
| **YTHDC2_rs75714066: G > C** | Allergy | 0,003 | 3,7 (1,6-8,7)^d^ |
|  |  |  |  |
| **ADAMTS17_rs72755233: G > A** | Pancreatitis | 0,002 | 5,5 (1,9-16,5)^d^ |
| **MYBBP1A_rs3809849: G > C** | Pancreatitis | 0,003 | 7,3 (2,0-26,9)^d^ |
| **SPECC1_rs9908032: C > G** | Pancreatitis | 0,002 | 4,2 (1,7-10,5)^a^ |
|  |  |  |  |
| **PKD2L1_rs6584356: C > A** | Thrombosis | 0,05 | 5,1 (1,0-26,1)^d^ |
| **RIN3_rs3742717: C > T** | Thrombosis | 0,01 | 13,9 (1,7-115,3)^r^ |
| **SPEF2_rs34708521: G > A** | Thrombosis | 0,08 | 4,3 (0,8-22,3)^d^ |
| **SLC39A12_rs62619938: C > T** | Thrombosis | 0,004 | 5,8 (1,8-19,1)^a^ |
| **MPEG1_rs7926933: G > A** | Thrombosis | 0,02 | 5,2 (1,2-21,7)^d^ |
| **IL16_rs11556218: T > G** | Thrombosis | 0,02 | 6,0 (1,3-27,7)^d^ |
|  |  |  |  |
| **MYBBP1A_rs3809849: G > C** | Thrombosis | 0,01 | 12,1 (1,6-100,5)^r^ |
| **SPEF2_rs34708521: G > A** | Pancreatitis | 0,05 | 3,3 (1,0-10,8)^a^ |
| **IL16_rs11556218: T > G** | Pancreatitis | 0,03 | 3,4 (1,2-10,3)^a^ |
| **SNP** | **Survival** | **P** | **HR (95%-CI)** |
| **MYBBP1A_rs3809849: G > C** | Event Free | 0,007 | 3,8 (1,4-9,8)^r^ |
| **MYBBP1A_rs3809849: G > C** | *Overall | 0,002 | 7,6 (2,0-28,4)^r^ |

The regression models included genotypes and as covariates, age, sex, risk, DFCI protocol ASNase formulation Genotypes were coded according to genetic models presented in Table 2 (a: additive, d: dominant and r: recessive in OR column). OR, odds ratio; HR, hazard ratio.

**Supplemental Table S4. Combined-cohort analysis performed for SNPs with reproducible associations with pancreatitis.**

| **Gene_SNP Genotype** | **Complication** | | **OR (95%-CI)** | **P** | **Combined Model** | **Complication** | | **OR (95%-CI)** | **P** |
| --- | --- | --- | --- | --- | --- | --- | --- | --- | --- |
|  | **+** | **-** |  |  |  | **+** | **-** |  |  |
| **MYBBP1A_rs3809849: G > C** | | | | | | | | | |
| GG | 11  (32,4%) | 342  (65,0%) | 1 | 1 | GG | 11  (32,4%) | 342  (65,0%) | 1 | *Ref.* |
| GC | 22  (64,7%) | 160  (30,4%) | 4,3  (2,0-9,0) | 0.00008 | GC+CC | 23  (67,6%) | 184  (35,0%) | 3,9  (1,8-8,1) | **0.0003** |
| CC | 1  (2,9%) | 24  (4,6%) | 1,3  (0,2-10,5) | 1 |  |  |  |  |  |
| **SPEF2_rs34708521: G > A** | | | | | | | | | |
| GG | 18  (72,0%) | 396  (90,8%) | 1 | 1 | GG | 18 (72,0%) | 396  (90,8%) | 1 | *Ref.* |
| GA | 6  (24,0%) | 38  (8,7%) | 3,5  (1,3-9,3) | 0.02 | GA+AA | 7  (28,0%) | 40  (9,2%) | 3,8  (1,5-9,8) | **0.008** |
| AA | 1  (4%) | 2  (0,5%) | 11  (0,9-127) | 0.1 |  |  |  |  |  |
| **IL16_rs11556218: T > G** | | | | | | | | | |
| TT | 18  (66,7%) | 391  (85,4%) | 1 | 1 | TT | 18  (66,7%) | 391  (85,4%) | 1 | *Ref.* |
| TG | 8  (29,6%) | 61  (13,3%) | 2,8  (1,2-6,8) | 0.02 | TG+GG | 9  (33,3%) | 67  (14,6%) | 2,9  (1,3-6,8) | **0.02** |
| GG | 1  (3,7%) | 6  (1,3%) | 3,6  (0,4-31,6) | 0.3 |  |  |  |  |  |

The combined-cohort represents the pooled samples from the discovery and replication cohort (QcALL+DFCI) which gives rise to a cohort with a larger sample size of 584 patients. The SNPs are presented as a change from major to minor alleles. OR, odds ratio; CI, confidence interval. Analysis in both co-dominant and dominant models are presented.
